# Machine Learning Approaches for Predicting Psoriatic Arthritis Risk Using Electronic Medical Records: Population-Based Study

**DOI:** 10.2196/39972

**Published:** 2023-03-28

**Authors:** Leon Tsung-Ju Lee, Hsuan-Chia Yang, Phung Anh Nguyen, Muhammad Solihuddin Muhtar, Yu-Chuan Jack Li

**Affiliations:** 1 Graduate Institute of Biomedical Informatics Taipei Medical University Taipei Taiwan; 2 Graduate Institute of Clinical Medicine Taipei Medical University Taipei Taiwan; 3 Department of Dermatology Taipei Medical University Hospital Taipei Medical University Taipei Taiwan; 4 Department of Dermatology, School of Medicine College of Medicine Taipei Medical University Taipei Taiwan; 5 Graduate Institute of Data Science Taipei Medical University Taipei Taiwan; 6 Department of Healthcare Information and Management Ming Chuan University Taoyuan Taiwan; 7 Department of Dermatology Taipei Municipal Wanfang Hospital Taipei Medical University Taipei Taiwan

**Keywords:** convolutional neural network, deep learning, machine learning, prediction model, psoriasis, psoriatic arthritis, temporal phenomic map, electronic medical records

## Abstract

**Background:**

Psoriasis (PsO) is a chronic, systemic, immune-mediated disease with multiorgan involvement. Psoriatic arthritis (PsA) is an inflammatory arthritis that is present in 6%-42% of patients with PsO. Approximately 15% of patients with PsO have undiagnosed PsA. Predicting patients with a risk of PsA is crucial for providing them with early examination and treatment that can prevent irreversible disease progression and function loss.

**Objective:**

The aim of this study was to develop and validate a prediction model for PsA based on chronological large-scale and multidimensional electronic medical records using a machine learning algorithm.

**Methods:**

This case-control study used Taiwan’s National Health Insurance Research Database from January 1, 1999, to December 31, 2013. The original data set was split into training and holdout data sets in an 80:20 ratio. A convolutional neural network was used to develop a prediction model. This model used 2.5-year diagnostic and medical records (inpatient and outpatient) with temporal-sequential information to predict the risk of PsA for a given patient within the next 6 months. The model was developed and cross-validated using the training data and was tested using the holdout data. An occlusion sensitivity analysis was performed to identify the important features of the model.

**Results:**

The prediction model included a total of 443 patients with PsA with earlier diagnosis of PsO and 1772 patients with PsO without PsA for the control group. The 6-month PsA risk prediction model that uses sequential diagnostic and drug prescription information as a temporal phenomic map yielded an area under the receiver operating characteristic curve of 0.70 (95% CI 0.559-0.833), a mean sensitivity of 0.80 (SD 0.11), a mean specificity of 0.60 (SD 0.04), and a mean negative predictive value of 0.93 (SD 0.04).

**Conclusions:**

The findings of this study suggest that the risk prediction model can identify patients with PsO at a high risk of PsA. This model may help health care professionals to prioritize treatment for target high-risk populations and prevent irreversible disease progression and functional loss.

## Introduction

Psoriasis (PsO) and psoriatic arthritis (PsA) are multiorgan inflammatory diseases with a similar pathophysiology. The prevalence of PsO ranges from 0.09% to 11.4% worldwide [1,2], and PsA occurs in approximately 12.7%-30% of patients with PsO [3,4]. Patients with PsO develop PsA in approximately 7-12 years [5,6]. Irreversible joint deformities develop within 5-10 years in 30%-40% of patients with PsA and adversely affect many aspects of patients’ lives. According to the meta-analysis of Villani et al [7], PsA was undiagnosed in 15.5% of patients with PsO. The prediction of PsA before irreversible joint or bone damage is crucial. Early control of the inflammatory burden can reduce the severity of comorbidities [8]. Therefore, a tool for predicting PsA could assist physicians in promptly intervening to reduce inflammation.

Machine learning (ML) algorithms have been used to diagnose diseases [9], predict disease progression [10], and evaluate responses to treatment [11]. Convolutional neural networks (CNNs) are types of ML suitable for working with spatial data, such as images. CNNs can enhance diagnostic capabilities and support decision-making based on medical imaging in the fields of dermatology, pathology, and radiology because of their strong image recognition capabilities. The clinical applications of CNNs include lesion detection and evaluation as well as disease prediction by analyzing electronic medical records (EMRs) [12]. Cheng et al [13] used a temporal matrix with time as one dimension and diagnostic codes as the other and validated the effectiveness of the proposed CNN model in predicting congestive heart failure and chronic obstructive pulmonary disease using EMRs. Wang et al [10] visualized EMRs by importing timeline information to increase the dimension of input data (diagnostic and prescription codes). A risk prediction model was established to predict incident nonmelanoma skin cancer.

ML has been increasingly applied to PsO and PsA, and its use is not limited to the diagnosis or differentiation of cutaneous psoriatic lesions in an image or the assessment of cutaneous lesion severity. Patrick et al [3] used a genetic signature to analyze the differences between PsA and cutaneous PsO and constructed a model to predict the risk of PsA in patients with PsO before the appearance of joint symptoms, with an area under the receiver operating characteristic curve (AUROC) of 0.82. They combined statistical methods and ML techniques to identify genetic differences between various PsO subtypes and conducted a personalized PsO subtype risk assessment. However, it requires performing genome-wide association studies, which are expensive and time-consuming for each patient.

No tool, however, is available to predict PsA in the preclinical stages, especially in cases without the typical cutaneous or nail presentation of PsO. Haroon et al [14] demonstrated that a delay in the intervention of more than 6 months from symptom onset can result in severe joint damage and poor prognosis. This study proposed a chronological EMR-based deep learning algorithm to predict the risk of PsA in patients with PsO. The model was built to provide an effective tool for dermatologists to screen patients with PsO at a high risk of PsA and may help physicians prevent or delay disease progression in the early stages.

## Methods

### Data Source

Data were obtained from Taiwan’s National Health Insurance Research Database (NHIRD), which contains data on the 2 million beneficiaries enrolled in Taiwan’s National Health Insurance (NHI). The database contains extensive real-world data, such as original claims data for reimbursement and the registration files of beneficiaries and health care facilities. Data from January 1, 1999, to December 31, 2013, were collected. The NHIRD provides information regarding NHI usage and medical care, including demographic characteristics, diagnostic and procedure codes, as well as prescription details.

### Ethics Approval

The research was reviewed and approved by the Institutional Review Board of Taipei Medical University (N201701027)

### Study Population and Design

This study was a case-control study (ratio of cases to controls: 1:4) and enrolled patients aged between 5 and 99 years who had at least 3 years (ie, 156 weeks) of records between January 1, 2002, and December 31, 2013.

The purpose of the PsA model was to distinguish patients susceptible to developing PsA from PsO. In this model, PsA was defined as patients with PsO who had at least two International Classification of Diseases, Ninth Revision, Clinical Modification (ICD-9-CM) code 696.0 (psoriatic arthropathy) diagnoses from outpatient visits or at least one from an admission claim. Patients without a diagnosis of PsO (ICD-9-CM code 696.1 or 696.8) and with a first-time diagnosis of PsO after PsA were excluded. The control group included patients with PsO (ICD-9-CM code 696.1 or 696.8) and excluded patients with PsA (ICD-9-CM code 696.8; Figure S1 in Multimedia Appendix 1).

The index date was the first date of PsA diagnosis. The index date of the control group was the last date for which medical records were available in the database. This study used 2.5 years (131 weeks) as the observation window and 0.5 years (25 weeks) as the prediction window. The medical information from the observation window was used to predict new-onset PsA 0.5 years in advance.

### Prediction Model Construction

This study used age, sex, ICD-9-CM diagnostic codes, and the Anatomical Therapeutic Chemical (ATC) prescription codes of the World Health Organization during the observation window to establish features. This study used 1098 (999+99) ICD-9-CM codes to translate the diagnosis of diseases and other health problems from text to code. The codes (999) were divided into 17 chapters on the basis of the cause and anatomy of the system and supplemented with V codes (99). In the ATC classification system, drugs are classified on the basis of their target organs or systems and their chemical, pharmacological, and therapeutic properties. A total of 830 drug categories were included in this study. The first 3 digits of the ICD-9-CM codes were used as diagnostic information as well as the first 5 characters of most of the ATC codes and the first 7 characters for medications, with “x” as the fifth character, were used as prescription information.

**Figure 1:**
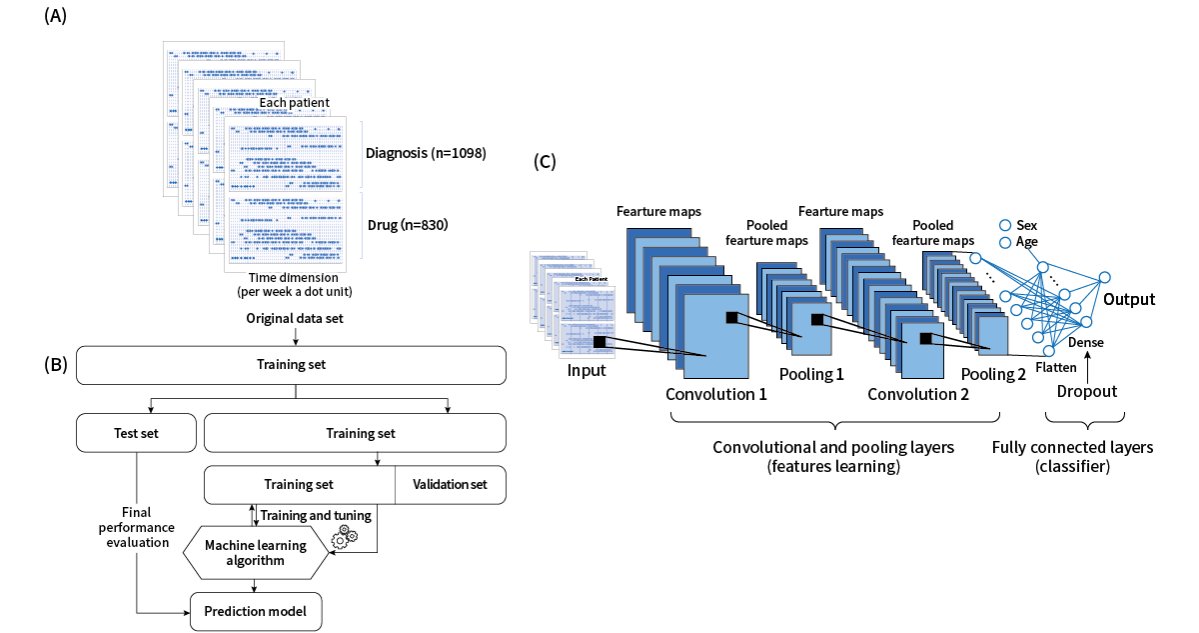
An overview of the prediction model for psoriatic arthritis. (A) Schematic of the temporal phenomic map. (B) Schematic of the machine learning classification framework. (C) Structure of the convolutional neural network.

Supervised CNN models were constructed to differentiate between the presence or absence of PsA as a binary classification problem. The input layer of the CNN model comprised EMR information arranged chronologically. Each patient had their own characteristic temporal phenomic map (TPM). The vertical axis was the diagnosis and drug records. The horizontal axis was the date of a hospital visit and drug prescription. Each dot in the TPM represented the number of times a certain diagnosis was established on a certain visit and the number of days a certain drug was prescribed; these values ranged from 0 to 1 after normalization (Figure 1A).

The original data set was divided into training and holdout sets at an 80:20 ratio (Figure 1B). Five-fold cross-validation was performed to train and evaluate the model, prevent problems such as overfitting, and decrease generalization error. Subsequently, 80% of the data were used for training, and 20% were used for testing; the process was executed 5 times to cover all the data. Finally, the holdout set was used to provide an unbiased evaluation of the final model fit on the training set. Figure 1C displays the CNN architecture. The hidden layers were (1) the convolutional layer, (2) the pooling layer, (3) the flatten layer, (4) the concatenation layer, (5) the dropout layer, and (6) the fully connected layer. The output was a normalized probability of PsA ranging from 0 to 1, which was then converted to crisp class labels. The CNN model consisted of several pairs of convolutional and pooling layers, which were converted to a flatten layer. Each convolutional layer was followed by an activation function that provided the nonlinear transformation capability required by the network. The flatten layer was concatenated with information regarding age and sex, and this was followed by the operation of the fully connected layers. The dropout layer was used for regularization to avoid overfitting. The activation function was applied to the last fully connected layer for classification. This study was performed on Keras (version 2.3.0; Google Inc) with the TensorFlow framework (backend; version 2.2; Google Inc) in a Python 3.7 environment (Google Colab; Google Inc).

### Model Evaluation

The AUROC, sensitivity, specificity, positive predictive value, and negative predictive value were calculated to evaluate the performance of the models [15]. AUROC was used to evaluate the predictive value of the model. The optimal discrimination threshold for AUROC was the point at which both sensitivity and specificity were maximized (sensitivity+specificity).

### Feature Importance

This study interpreted the deep learning algorithm by examining the relationship between features and model performance. An occlusion sensitivity analysis was performed to identify the most crucial parts of the TPM for the neural network’s classification [16]. Stepwise elimination was performed to examine AUROC loss and identify the crucial factors of the model. Features or groups of features were eliminated one by one in a stepwise manner as described in the literature to determine AUROC loss (Figure S2 in Multimedia Appendix 1). After the crucial features were identified, a logistic regression (LR) analysis was performed on the predictors and PsA. The dependence of PsA occurrence on the predictors was quantified using odds ratios.

### Statistical Analysis

Data were reported as means with SDs for the parametric variables. This study performed an LR analysis of PsA and its predictors. *P*<.05 was considered statistically significant. Statistical analysis was performed using SAS Enterprise Guide (version 7.1) and Enterprise Miner (version 14.3; SAS Institute Inc).

## Results

### Demographics of the Sampled Data Set

Table 1 lists the demographic information. The mean age was 42.66 (SD 17.21) years, and the PsA group comprised 266 (61.43%) men. In the randomly sampled control group, the mean age was 46.85 (SD 20.18) years, and the group comprised 989 (57.10%) men. The TPM of each patient consisted of 2.5 years of time-series medical records (ICD-9-CM codes for each visit and prescription medications obtained on each date). The annual number of ICD-9-CM codes multiplied by clinical visits per person was 32.6 in the PsA group and 40.1 in the control groups. The annual number of drugs multiplied by prescription days per person was 50.4 in the PsA group and 43.9 in the control groups.

**Table 1:** Demographics of the sampled data set.

Characteristics			Psoriatic arthritis group (n=443)		Control group (n=1772)
Age (years), mean (SD)^a^			42.66 (17.21)		46.85 (20.18)
Age (years), range (min-max)			5-95		7-94
**Sex, n (%)^a^**					
	Male	266 (61.43)		989 (57.10)	
	Female	177 (38.57)		783 (42.90)	
Total diagnosis (ICD-9-CM^b^), n (%)			520 (47.36)		695 (63.30)
Total medication (ATC^c^), n (%)			415 (50)		516 (62.17)
**Annual accumulation, n**					
	ICD-9-CM counts per person	32.6		40.1	
	Medications per person	50.4		43.9	

^a^*P*<.001.

^b^ICD-9-CM: International Classification of Diseases, Ninth Revision, Clinical Modification.

^c^ATC: Anatomical Therapeutic Chemical.

### The Architecture of the CNN Model

The model consisted of 8 hidden layers [17]. Figure S3 in Multimedia Appendix 1 displays the CNN architecture. The first layer was a convolutional layer with 32 filters in a 1×131 shape, where 131 represents the total number of weeks in 2.5 years, which was the x-axis of the input TPM. The second layer was the average pooling layer, which calculated the average for each patch of the feature map. The purpose of the pooling layer was (1) to reduce the number of parameters and dimensions of data and the computational cost by subsampling the input image and (2) to maintain feature invariance. The average pooling size was 2×2. The third layer was a convolutional layer with 2 filters in a 1×131 shape. The fourth layer was the max pooling layer. The max pooling size was 1×3. The fifth layer was a flatten layer, and the sixth layer was concatenated with information regarding sex and age. The seventh layer was a fully connected layer with 128 neurons. The eighth layer was a dropout layer with a dropout rate of 0.3 for the overfitting problem. One neuron represented the possibility of risk in the output layer with sigmoid activation.

Regarding the hyperparameters of the CNN, the epoch was set to 20 to obtain the optimal AUROC on the basis of the experimental results, and the batch size was 64. The learning rate was optimized by Adam. The activation functions of the convolutional and fully connected layers were reLU and LeakyReLU, and the activation function of the output layer was sigmoid. All patients in the training data set were randomly divided, with 80% for training and 20% for 5-fold cross-validation. Approximately 60 to 120 minutes were required to complete the 5-fold cross-validation.

### Performance of the CNN Model

**Figure 2:**
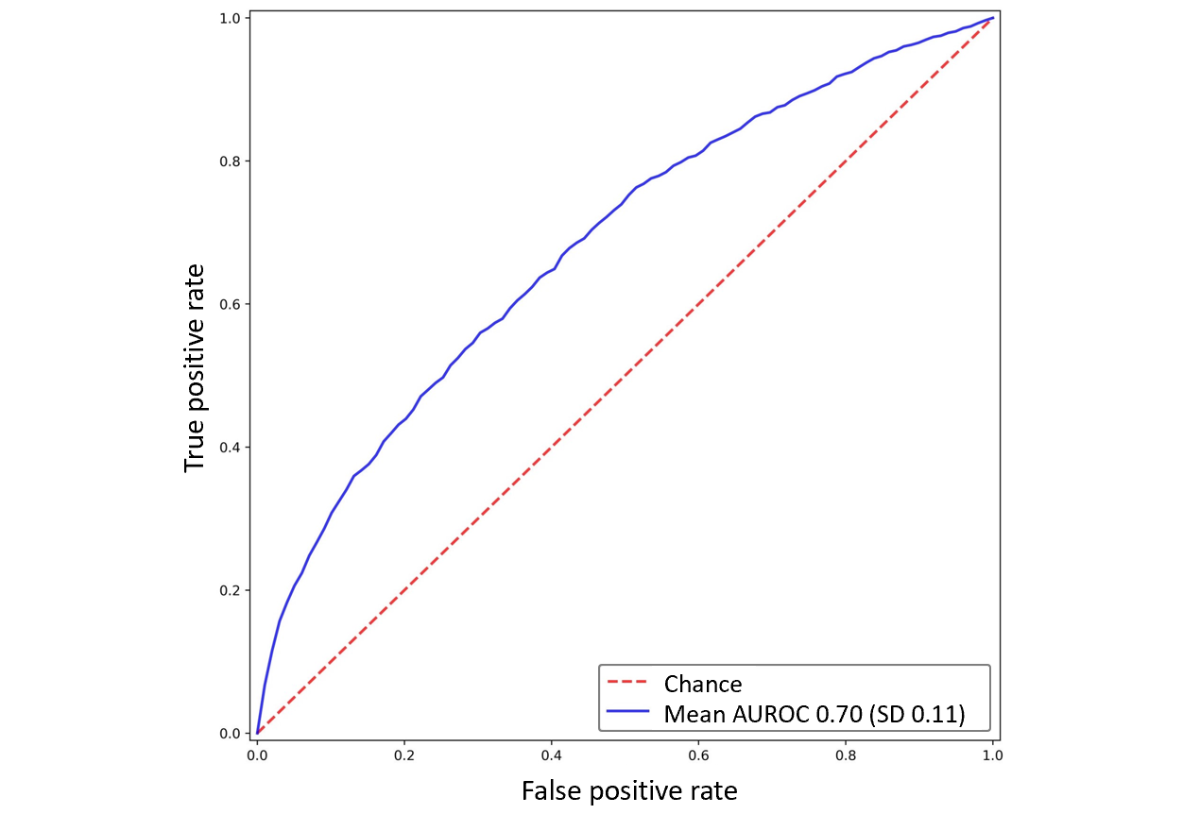
Receiver operating characteristic curve of the model using sequential medical records. AUROC: area under the receiver operating characteristic curve.

The PsA model predicted PsA with a mean AUROC of 0.70 (SD 0.11; 95% CI 0.559-0.833), a mean sensitivity of 0.80 (SD 0.11), a mean specificity of 0.60 (SD 0.04), a mean positive predictive value of 0.32 (SD 0.05), and a mean negative predictive value of 0.93 (SD 0.04; Table S1 in Multimedia Appendix 1). Figure 2 presents the receiver operating characteristic curve of the model. The risk probability score ranged from 0 (no disease) to 1 (disease). The optimal discrimination threshold was 0.429. Models with a case-control ratio of 1:10 and 10-fold cross-validation were used. However, their performance was not satisfactory, with a mean AUROC of 0.64 (SD 0.06; 95% CI 0.595-0.687; Figure S4 in Multimedia Appendix 1).

### Crucial Features of the CNN Models

An occlusion sensitivity analysis was performed to interpret the CNN model. This analysis was performed to identify the most crucial parts of the TPM for model classification. The features were identified by evaluating AUROC loss through stepwise elimination.

The AUROC loss of the PsA model ranged from −0.001% to −3.54%. Table S2 in Multimedia Appendix 1 presents the features with the strongest effects on the power of prediction (>2.02% loss). Age was the most crucial feature in the model, with an AUROC loss of −3.54%. Autoimmune connective tissue diseases (−2.20%); rheumatoid arthritis and other inflammatory polyarthritis (−2.10%); anxiety and depression (−2.07%); ankylosing spondylitis and other inflammatory spondylopathies (−2.07%); osteoporosis and pathologic fracture (−2.07%); atopic dermatitis (−2.04%); menopausal and postmenopausal disorders (−2.06%); as well as other chronic comorbidities, such as renal diseases (−2.10%), obesity and metabolic syndrome (−2.08%), dyslipidemia (−2.08%), cardiovascular disorders (−2.06%), diabetes (−2.03%), and hypertension (−2.03%), were identified as crucial features.

Medications such as disease-modifying antirheumatic drugs (DMARDs; −2.06%); methotrexate (−2.10%); azathioprine (−2.09%); acitretin (−2.09%); aminoquinolines like hydroxychloroquine and chloroquine (−2.06%); calcineurin inhibitors like ciclosporin (−2.06%); selective immunosuppressants like leflunomide and tofacitinib (−2.06%); and sulfasalazine (−2.02%) were identified as crucial features. Topical medications, such as tars (−2.82%), corticosteroids, and vitamin D analogues—calcipotriol (−2.77%) and calcitriol (−2.09%)—were identified as crucial features.

## Discussion

### Principal Results

A CNN model was constructed to identify patients with PsO at a high risk of PsA 6 months in advance using chronological EMRs, with an AUROC of 0.70. Early intervention can prevent PsA, especially in patients with PsO. This model used prescription and medication EMR data for prediction and did not require other information, such as nail lesions, severity, area of cutaneous lesions, or family history. In addition, potential predictive features, such as comorbidities and medications, were analyzed. This PsA prediction model may help physicians to identify high-risk groups and intervene before irreversible disease progression and functional loss.

PsA is a severe and often irreversible comorbidity of PsO that substantially affects patients’ quality of life. Patrick et al [3] constructed an ML pipeline to distinguish patients with PsA from those with PsO on the basis of genetic background. The AUROC was 0.82 in the cross-validation and testing when 200 genetic markers were used. Although the AUROC of our PsA model was 0.70 for the test set, this study only used the easy-to-obtain and simple ICD-9-CM diagnostic and ATC medication codes in EMRs to construct the model rather than conducting a genome-wide association study for each patient, which is expensive and time-consuming.

Mease et al [18] indicated that 41% of patients with PsA had not received their diagnosis at dermatology clinics. Villani and colleagues [7] estimated that 15.5% of patients with PsO have undiagnosed PsA. The high prevalence of undiagnosed PsA in patients with PsO should remind dermatologists of the criticality of screening all patients with PsO for PsA. Mease et al [19] evaluated 3 PsA screening questionnaires: the Psoriasis and Arthritis Screening Questionnaire, the Psoriasis Epidemiology Screening Tool, and the Toronto Psoriatic Arthritis Screening Questionnaire. The negative predictive value of our PsA model (0.93) was higher than those of the screening tools in Mease et al (0.83-0.91) [18], but the sensitivity was similar (0.80 vs 0.67-0.84). The specificity of our PsA model (0.60) was also similar to that of Mease et al (0.64-0.75) [18]. Therefore, the prediction models in this study are useful screening tools for detecting probable or subclinical PsA.

An occlusion sensitivity analysis is a simple technique used to determine how a CNN makes a classification [16]. The output of the model can be observed by occluding a part of the image to determine the blocks in the image that are more crucial for model classification. This study used a similar technique for the TPM-based ML model by removing one or more diagnostic or medication codes in a stepwise manner and observing the changes in the AUROC. When a crucial part of a TPM is removed, the AUROC of the prediction should decrease substantially. Most of the decisive factors in this study were consistent with those identified in the literature.

The comorbidities resulting in the greatest AUROC loss (>2.03%) when removed one code at a time were the diffuse diseases of systemic lupus erythematosus, rheumatoid arthritis, vitiligo, alopecia, diabetes, hypertension, cardiovascular disease, dyslipidemia, metabolic syndrome, kidney disease, and psychosis, which confirmed the multisystemic nature of PsA [2,4,20].

The drugs resulting in the greatest AUROC loss (>2.02%) when removed one code at a time were topical corticosteroids, topical vitamin D analogs, tars, methotrexate, acitretin, ciclosporin, leflunomide, and sulfasalazine, indicating that the use of topical steroids and DMARDs for PsO strongly affected the prediction of the models. High-potency topical steroids and DMARDs are usually reserved for severe and refractory PsO, and these results indicate that more severe skin symptoms are associated with a higher risk of PsA [5].

Because the results from ML are not necessarily based on clinical evidence, physician interpretation is crucial for practical implementation. Studies have performed regression analyses to identify the correlation between dependent and independent variables [21]. LR is performed to obtain ORs in cases with more than 1 explanatory variable to mitigate the effect of confounding factors, [22] but correlation does not imply causation in LR. Deep learning techniques are particularly suited to complex data sets with nonlinear solutions, especially in high-dimensionality data sets. In this study, the CNN model weighted whole factors, including chronological sequence, at the same time for prediction; thus, it could not provide reliable statistical inferences of associations because of the black box nature. However, even a feature without a statistically significant correlation can function as a predictor in the model. Although ML can assist physicians in making diagnoses and treatment plans, it cannot replace physicians’ decision-making process, which is based on clinical evidence and experience [23].

### Limitations

This study had several potential limitations. First, this study was retrospective in nature. Because of the lack of prospective and external validation data, the utility of these models in clinical scenarios for real-world applications must be evaluated. Second, the optimal structure and hyperparameters of CNNs trained on certain data and the number of cases required to train models varied from task to task [24]. Third, the data in this study were from 1999 to 2013; the absence of PsA in this 15-year period did not mean that the patients would not develop PsA in the future. Fourth, although Taiwan’s NHI claims data are particularly valuable because of their standardization, representativeness, and comprehensiveness, we should keep in mind that the claims data are intended for administrative purposes and lack data on examination results, disease severity, and health behavior [25].

### Conclusions

PsA causes irreversible joint damage that severely affects patients’ daily functions and quality of life and is a burden on medical resources. No simple and reliable predictive tools or criteria are available to help physicians intervene early before joint symptoms appear. In addition, PsA is often indistinguishable from other types of inflammatory arthritis, such as rheumatoid arthritis and ankylosing spondylitis, if no skin or nail symptoms are present. Because most skin symptoms of PsO appear before joint symptoms, dermatologists must be diligent in identifying and treating patients with PsA. This study visualized EMRs and temporal information as TPMs and created a computer vision model using CNNs. Our prediction model achieved good performance for predicting PsA risk using standardized and population-level claim data. The predictive models may be used as a screening tool to assist physicians in risk stratification and identifying psoriatic patients with a high risk of PsA.

## Appendix

Multimedia Appendix 1 Supplementary materials.

## Abbreviations

ATC: Anatomical Therapeutic Chemical
AUROC: area under the receiver operating characteristic curve
CNN: convolutional neural network
DMARD: disease-modifying antirheumatic drug
EMR: electronic medical record
ICD-9: International Classification of Disease, Ninth Revision
LR: logistic regression
ML: machine learning
PsA: psoriatic arthritis
PsO: psoriasis
TPM: temporal phenomic map
