# Supplementary material for: Machine Learning Approaches for Predicting Psoriatic Arthritis Risk Using Electronic Medical Records: Population-Based Study
Source: J Med Internet Res. 2023 Mar 28;25:e39972. doi: 10.2196/39972 (PMC10055385; doi:10.2196/39972)
Supplement: Multimedia Appendix 1 [file jmir_v25i1e39972_app1.docx]

**List of Supplementary Materials**

[**Figure S1.** Study design and flowchart of patient selection. 1](#_Toc89637916)

[**Figure S2.** Occlusion sensitivity analysis. 2](#_Toc89637918)

[**Figure S3.** Algorithm flowchart of the convolutional neural network. 3](#_Toc89637919)

[**Figure S4.** Receiver operating characteristic curve. 4](#_Toc89637920)

[**Table S1.** Performance of convolutional neural network 5](#_Toc89637921)

[**Table S2.** Crucial features for prediction of PsA 6](#_Toc89637922)


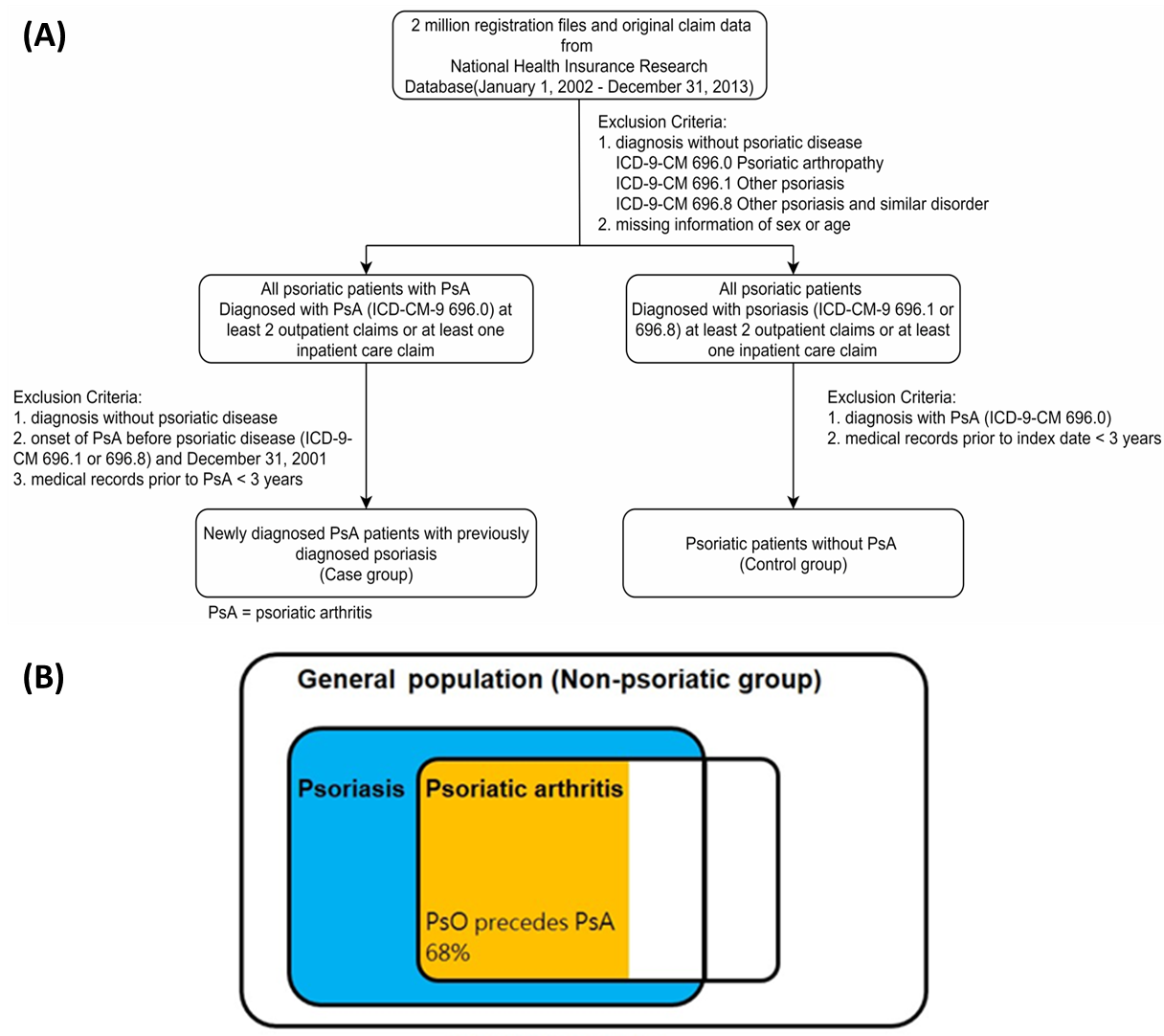


Figure S1. A Study design and flowchart of patient selection. B The relationship between the case group (yellow) and the control group (blue).


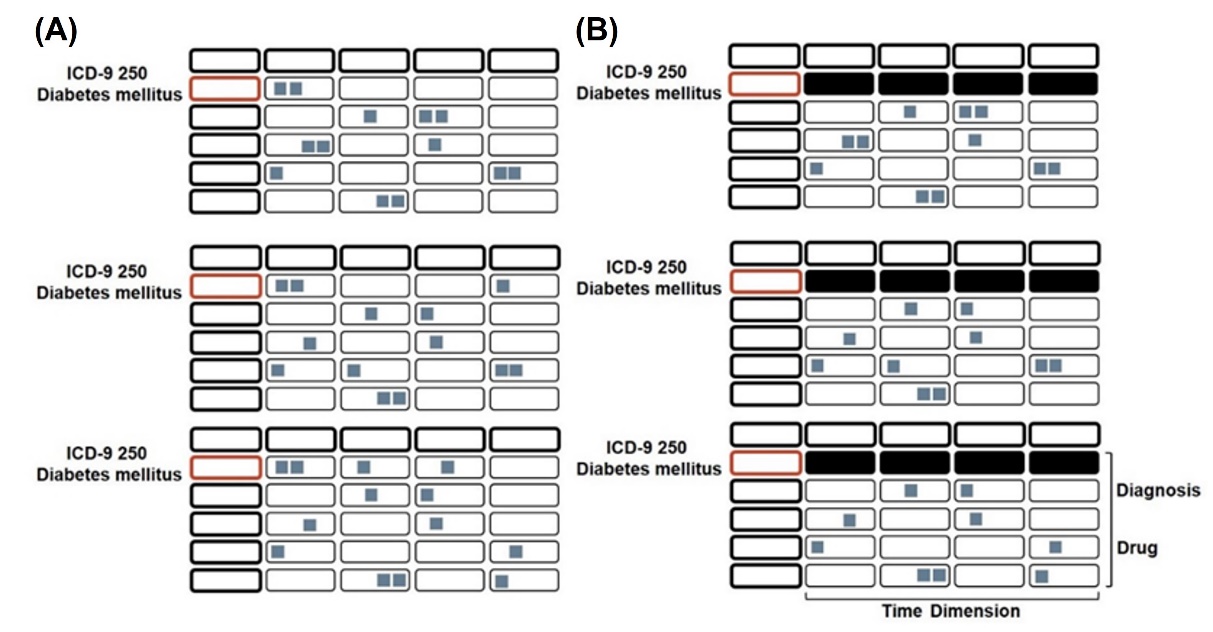


Figure S2. Occlusion sensitivity analysis.


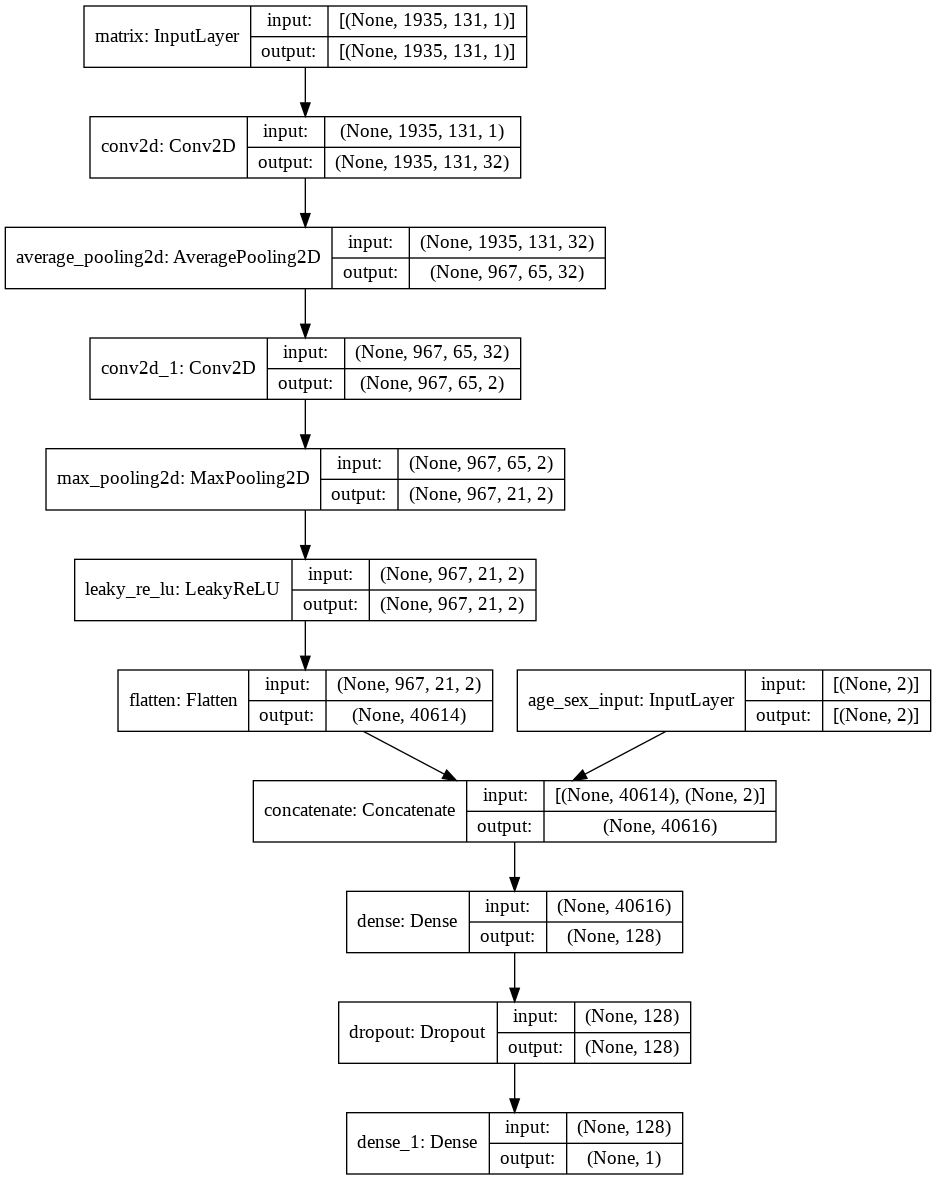


Figure S3. Algorithm flowchart of the convolutional neural network.


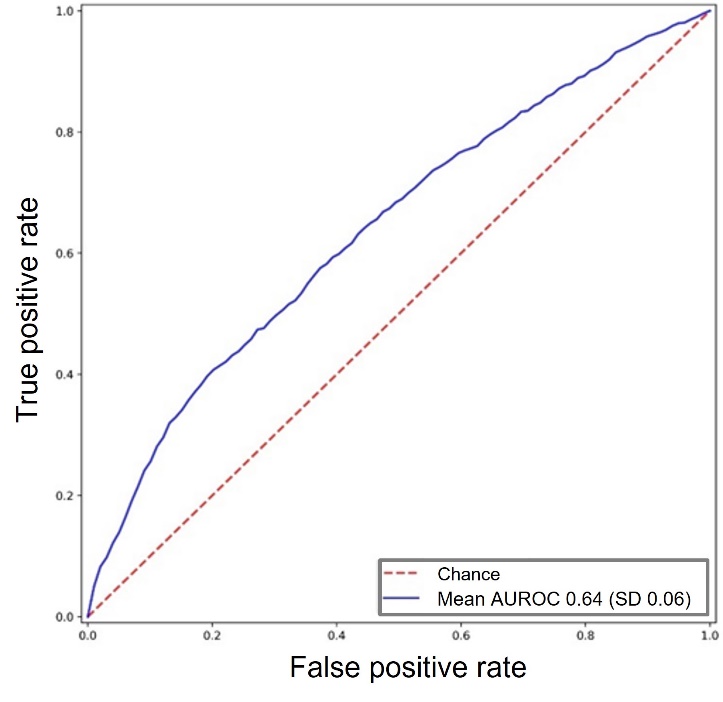


Figure S4. Receiver operating characteristic curve.

Table S1. Performance of convolutional neural network

|  | **Mean** | **SD** |
| --- | --- | --- |
| **AUROC** | 0.70 | 0.11 |
| **Sensitivity** | 0.80 | 0.11 |
| **Specificity** | 0.60 | 0.04 |
| **PPV** | 0.32 | 0.05 |
| **NPV** | 0.93 | 0.04 |

AUROC: area under the receiver operating characteristic; PPV: positive predictive value; NPV: negative predictive value.

Table S2. Crucial features for prediction of psoriatic arthritis

| **Feature** | **AUROC**  **(Loss %)^a^** | **Odds ratio**  **(*P* value)** |
| --- | --- | --- |
| All features included | 0.696 |  |
| Age | 0.671 (-3.54) | 0.993 (.069) |
| Sex | 0.674 (-3.18) | 0.955 (.480) ^d^ |
| **Comorbidities (*ICD-9-CM* code)** | | |
| Autoimmune connective tissue disease, e.g., systemic lupus erythematosus, Sicca syndrome and dermatomyositis (710) | 0.681 (-2.20) | 1.051 (.188) |
| Other disorders of skin and subcutaneous tissue, e.g., vitiligo (709) | 0.681 (-2.11) | 0.998 (.952) |
| Rheumatoid arthritis and other inflammatory polyarthropathies (714) | 0.681 (-2.10) | 1.017 (.599) |
| Renal diseases, e.g., glomerulonephritis, nephrotic syndrome and chronic kidney disease (580~588) | 0.681 (-2.10) | 0.988 (.390) |
| Gout (274) | 0.681(-2.09) | 1.031 (.079) |
| Obesity and metabolic syndrome (277, 278) | 0.682 (-2.08) | 1.009 (.929) |
| Disorders of lipid metabolism, e.g., mixed hyperlipidemia (272) | 0.682 (-2.08) | 0.988 (.515) |
| Diseases of hair and hair follicles, e.g., alopecia areata, telogen effluvium and other alopecia (704) | 0.682 (-2.07) | 0.977 (.570) |
| Other disorders of bone and cartilage, e.g., osteoporosis and pathologic fracture (733) | 0.682 (-2.07) | 1.096 (.011) |
| Ankylosing spondylitis and other inflammatory spondylopathies (720) | 0.682 (-2.07) | 1.119 (.018) |
| Anxiety and depression (296, 300, 309, 311) | 0.682 (-2.07) | 0.991 (.344) |
| Menopausal and postmenopausal disorders (627) | 0.682 (-2.06) | 1.040 (.212) |
| Cardiovascular disorders, e.g., myocardial infarction and angina pectoris (410~414, 440) | 0.682 (-2.06) | 0.978 (.258) |
| Atopic dermatitis and related conditions (691) | 0.682 (-2.04) | 0.956 (.235) |
| Diabetes mellitus (250) | 0.682 (-2.03) | 0.991 (.447) |
| Hypertension (401~405) | 0.682 (-2.03) | 0.976 (.037) |
| **Drug (ATC code)** | | |
| Tars ^b^ (D05AA) | 0.676 (-2.82) | 1.005 (<.001) |
| Calcipotriol ^b^ (D05AX02) | 0.677 (-2.77) | 1.008 (<.001) |
| Corticosteroids, moderately potent ^b^ (group II) (D07AB) | 0.681 (-2.12) | 1.003 (.406) |
| Folic acid analogues, e.g. methotrexate (L01BA) | 0.681 (-2.10) | 1.002 (.083) |
| Retinoids for treatment of psoriasis, e.g., acitretin (D05BB) | 0.681 (-2.09) | 0.999 (.697) |
| Calcitriol ^b^ (D05AX03) | 0.681 (-2.09) | 1.000 (.912) |
| Azathioprine (L04AX01) | 0.681 (-2.09) | 1.004 (.141) |
| Antigout drugs, e.g. allopurinol (M04AA) | 0.681 (-2.07) | 0.998 (.245) |
| Calcineurin inhibitors, e.g. ciclosporin (L04AD) | 0.682 (-2.06) | 1.003 (.261) |
| Disease-modifying antirheumatic drugs (DMARDs) ^c^ | 0.682 (-2.06) | 1.001 (.223) |
| Aminoquinolines, e.g. hydroxychloroquine and chloroquine (P01BA) | 0.682 (-2.06) | 0.996 (.042) |
| Selective immunosuppressants, e.g. leflunomide and tofacitinib (L04AA) | 0.682 (-2.06) | 0.995 (.134) |
| Aminosalicylic acid and similar agents, e.g., sulfasalazine (A07EC) | 0.682 (-2.02) | 1.000 (.872) |

^a^ The AUROC loss of the convolutional neural network when a feature was removed.
(AUROC_all features included_ − AUROC_selected feature removed_)/AUROC_all features included_

^b^ Topical medication

^c^ Disease-modifying antirheumatic drugs (D05BB, L01BA, L04AD, L04AA, A07EC)

^d^ Odds ratio for women
